# Supplementary material for: Assessment of Compaction, Temperature, and Duration Factors for Packaging and Transporting of Sterile Male Aedes aegypti (Diptera: Culicidae) under Laboratory Conditions
Source: Insects. 2022 Sep 17;13(9):847. doi: 10.3390/insects13090847 (PMC9501006; doi:10.3390/insects13090847)
Supplement: Supplementary file 1 [file insects-13-00847-s001.zip › insects-1858035-supplementary.pdf]

Table S1. Mean longevity of gamma-sterilized male *Ae. aegypti* post-treatment by density, temperature, and duration factors.

Treatments (Density and Duration)		Mean Longevity±SE (95% CI) (days) 7 °C	14 °C	21 °C	28 °C
*Control 1 (unirradiated–unpacked)		12.43±0.81 (10.84-14.02)$_{eA}$	12.81±0.8 (11.25-14.38)$_{dA}$	12.27±0.73 (10.84-13.7)$_{dA}$	13.79±0.89 (12.05-15.53)$_{eA}$
*Control 2 (irradiated–unpacked)		8.49±0.6 (7.32-9.67)$_{dA}$	8.79±0.57 (7.66-9.91)$_{cA}$	9.48±0.68 (8.14-10.82)$_{cA}$	10.39±0.67 (9.07-11.7)$_{eA}$
Density 40 males/2 mL	3 h	6.87±0.43 (6.03-7.7)$_{cdA\beta}$	6.44±0.46 (5.53-7.35)$_{bA\beta}$	7.77±0.49 (6.82-8.73)$_{cA\beta}$	8.04±0.73 (6.6-9.48)$_{dA\alpha}$
	6 h	5.53±0.42 (4.71-6.36)$_{bA\alpha}$	5.48±0.39 (4.72-6.24)$_{abA\beta}$	5.24±0.37 (4.52-5.96)$_{bA\beta}$	5.64±0.42 (4.81-6.47)$_{cdA\beta}$
	12 h	5.73±0.38 (4.99-6.47)$_{bcA\gamma}$	4.52±0.32 (3.89-5.15)$_{aA\alpha}$	4.97±0.38 (4.23-5.72)$_{bA\beta}$	4.56±0.29 (3.99-5.12)$_{bcA\beta}$
	24 h	5.16±0.34 (4.5-5.82)$_{bB\alpha}$	5.96±0.47 (5.05-6.87)$_{abB\beta}$	5.48±0.37 (4.76-6.2)$_{bB\beta}$	3.85±0.32 (3.23-4.48)$_{abA\beta}$
	48 h	3.59±0.18 (3.23-3.94)$_{aB\alpha}$	6.72±0.49 (5.75-7.69)$_{bC\gamma}$	3.28±0.283 (2.72-3.84)$_{aAB\beta}$	2.91±0.27 (2.38-3.43)$_{aA\beta}$
*Control 1 (unirradiated–unpacked)		12.43±0.81 (10.84-14.02)$_{dA}$	12.81±0.8 (11.25-14.38)$_{cA}$	12.27±0.73 (10.84-13.7)$_{eA}$	13.79±0.89 (12.05-15.53)$_{eA}$
*Control 2 (irradiated–unpacked)		8.49±0.6 (7.32-9.67)$_{cA}$	8.79±0.57 (7.66-9.91)$_{bA}$	9.48±0.68 (8.14-10.82)$_{dA}$	10.39±0.67 (9.07-11.7)$_{eA}$
Density 80 males/2 mL	3 h	5.59±0.4 (4.8-6.38)$_{abA\beta}$	5.73±0.41 (4.93-6.53)$_{aA\beta}$	5.21±0.39 (4.45-5.98)$_{cA\alpha}$	6.45±0.54 (5.4-7.51)$_{dA\alpha}$
	6 h	4.67±0.35 (3.99-5.35)$_{abA\alpha}$	5.52±0.38 (4.77-6.27)$_{aA\beta}$	4.71±0.35 (4.01-5.4)$_{cA\beta}$	4.84±0.36 (4.13-5.55)$_{cdA\beta}$
	12 h	4.39±0.32 (3.75-5.02)$_{aA\beta}$	5.05±0.39 (4.28-5.82)$_{aA\alpha}$	4.43±0.32 (3.79-5.06)$_{cA\beta}$	4.33±0.26 (3.82-4.85)$_{cA\beta}$
	24 h	5.47±0.31 (4.85-6.08)$_{bB\alpha}$	5.89±0.42 (5.08-6.71)$_{aB\beta}$	2.8±0.25 (2.32-3.28)$_{bA\alpha}$	3.07±0.29 (2.51-3.63)$_{bA\beta}$
	48 h	4.24±0.29 (3.66-4.82)$_{aB\alpha}$	5.27±0.48 (4.32-6.21)a$_{B\beta}$	1.77±0.18 (1.43-2.12)$_{aA\alpha}$	2.19±0.3 (1.61-2.77)$_{aA\alpha}$
*Control 1 (unirradiated–unpacked)		12.43±0.81 (10.84-14.02)$_{eA}$	12.81±0.8 (11.25-14.38)$_{dA}$	12.27±0.73 (10.84-13.7)$_{dA}$	13.79±0.89 (12.05-15.53)$_{dA}$
*Control 2 (irradiated–unpacked)		8.49±0.6 (7.32-9.67)$_{dA}$	8.79±0.57 (7.66-9.91)$_{cA}$	9.48±0.68 (8.14-10.82)$_{cA}$	10.39±0.67 (9.07-11.7)$_{dA}$
Density 120 males/2 mL	3 h	4.12±0.5 (3.14-5.1)$_{abA\alpha}$	4.32±0.47 (3.39-5.25)$_{abA\alpha}$	6.47±0.34 (5.8-7.13)$_{cB\alpha\beta}$	5.57±0.43 (4.74-6.41)$_{cB\alpha}$
	6 h	4.05±0.31 (3.44-4.66)$_{bcB\alpha}$	4.11±0.48 (3.16-5.06)$_{abAB\alpha}$	3.19±0.19 (2.81-3.56)$_{bAB\alpha}$	3.36±0.34 (2.69-4.03)$_{bA\alpha}$
	12 h	3.05±0.3 (2.47-3.64)$_{aA\alpha}$	4.93±0.46 (4.03-5.83)$_{bB\alpha}$	3.32±0.28 (2.77-3.87)$_{bA\alpha}$	3.39±0.29 (2.81-3.96)$_{bA\alpha}$
	24 h	4.84±0.3 (4.24-5.44)$_{cC\alpha}$	4.43±0.51 (3.43-5.43)$_{abB\alpha}$	2.92±0.26 (2.41-3.43)$_{bB\alpha}$	1.97±0.23 (1.52-2.42)$_{aA\alpha}$
	48 h	3.93±0.27 (3.41-4.46)$_{bcC\alpha}$	3.23±0.39 (2.46-3.99)$_{aB\alpha}$	1.48±0.14 (1.2-1.76)$_{aA\alpha}$	1.43±0.14 (1.16-1.7)$_{aA\alpha}$

*Control 1 and control 2 were not packed and exposed to temperature treatment. The same lowercase, uppercase, and symbol indicate no significant difference within the same density and temperature, the same duration and density, and the same duration and temperature, respectively (Log-rank and Pairwise Kruskal–Wallis tests, $p = 0.05$).
